# Utility of cone beam computed tomography for rare temporal bone lesion: A case report

**DOI:** 10.1016/j.radcr.2024.07.037

**Published:** 2024-08-03

**Authors:** Fernando Ahumada, Alejandro Jose Quiroz Alfaro, Orlando Diaz

**Affiliations:** a Department of Neuroradiology, The Houston Methodist Hospital, Houston, 6565 Fannin St, Houston, TX 77030, USA; b Internal Medicine, North Mississippi Medical Center, Tupelo, 830 South Gloster Street, Tupelo, MS 38801, USA

**Keywords:** Magnetic resonance imaging, Cone beam computed tomography, Cerebellopontine angle, Temporal bone mass, Central nervous system, Cerebral angiography

## Abstract

This case report aims to describe the clinical presentation, imaging findings, diagnostic challenges, and management of a patient with a cerebellopontine angle lesion. A 63-year-old woman presented with progressive headaches, tinnitus, right ear pressure, and dizziness. Initial imaging studies (computed tomography and magnetic resonance imaging) suggested either a thrombosed aneurysm or a lipoma. However, advanced imaging with cone beam computed tomography provided a definitive diagnosis of temporal bone exostosis. This case highlights the importance of cone beam computed tomography in diagnosing complex intracranial lesions due to its superior spatial resolution and lower radiation dose.

## Introduction

The cerebellopontine angle (CPA) can be affected by a diverse variety of lesions with a wide range of clinical manifestations; schwannomas and meningiomas are the most common types of lesions involving the CPA and account for 8.9% and 33.8% of central nervous system tumors, respectively [1], [2].

This article presents a case of a patient with a lesion in the cerebellopontine angle that was initially thought to be an aneurysm or a small lipoma. The case's significance is that the initial images suggested a particular diagnosis, but more advanced imaging techniques, such as cone beam CT (CBCT), were used to diagnose the patient more accurately. This highlights the importance of using advanced imaging techniques to improve precision in diagnosis.

## Case report

A 63-year-old female patient presented with a 5-week history of progressive, severe headaches accompanied by tinnitus, pressure in her right ear, and dizziness. The headaches particularly worsened during prolonged periods of walking. Her physical examination, including auditory and vestibular function tests, was unremarkable. The patient was treated with analgesics for the headache, antiemetics, and antihistamines for dizziness.

**Fig. 1:**
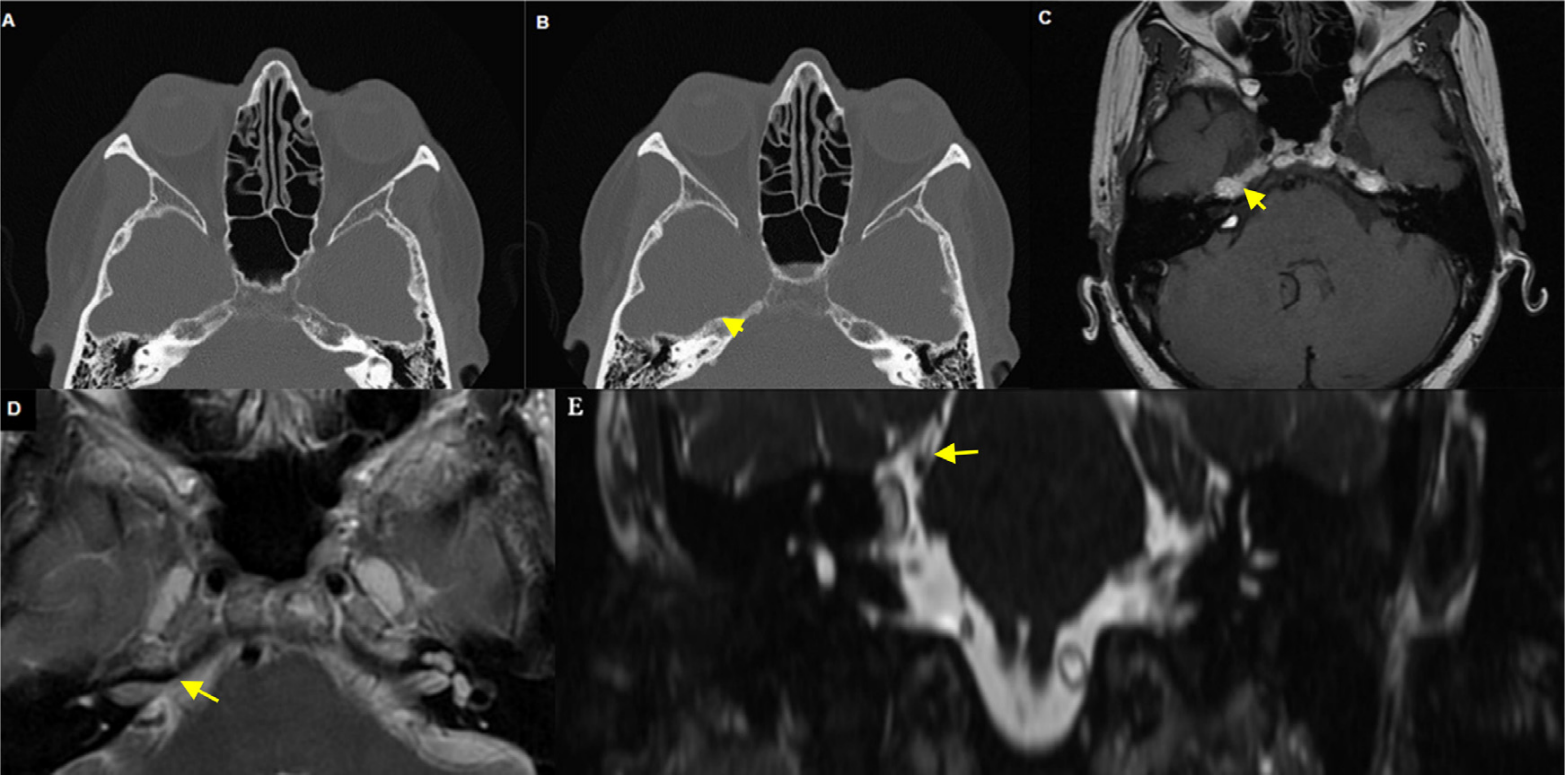
(A) Axial CT scan was normal, (B) Retrospectively showed small bone protrusion in the superior aspect of the right temporal bone (C and D) MRI Axial T1-T2 without contrast showed 5 mm lesion at the cerebellopontine (CP) angle, posterior and superior the internal auditory canal. The lesion appears hyperintense in the center surrounded hypointense halo. (E) Coronal MRI T2 image illustrates the relationship with the internal auditory canal.

**Fig. 2:**
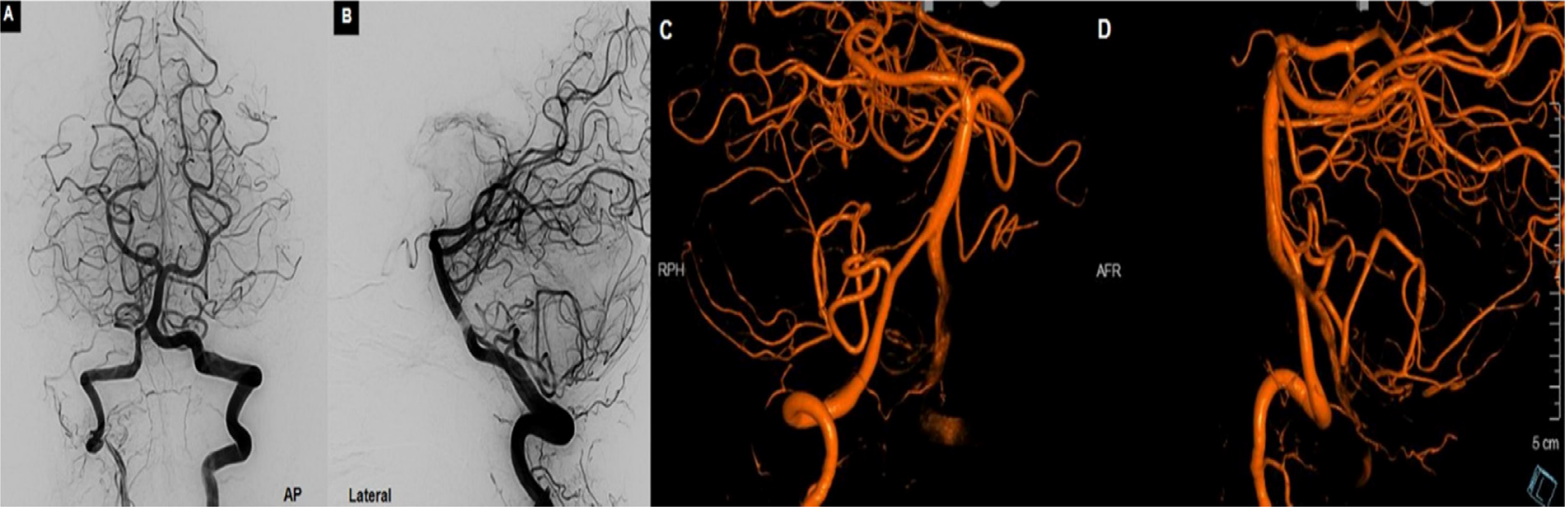
Cerebral angiography right vertebral artery injection AP (A), lateral view (B) and Tridimensional images (C)(D) indicate no evidence of aneurysm. AICA and PICA appear within normal limit.

**Fig. 3:**
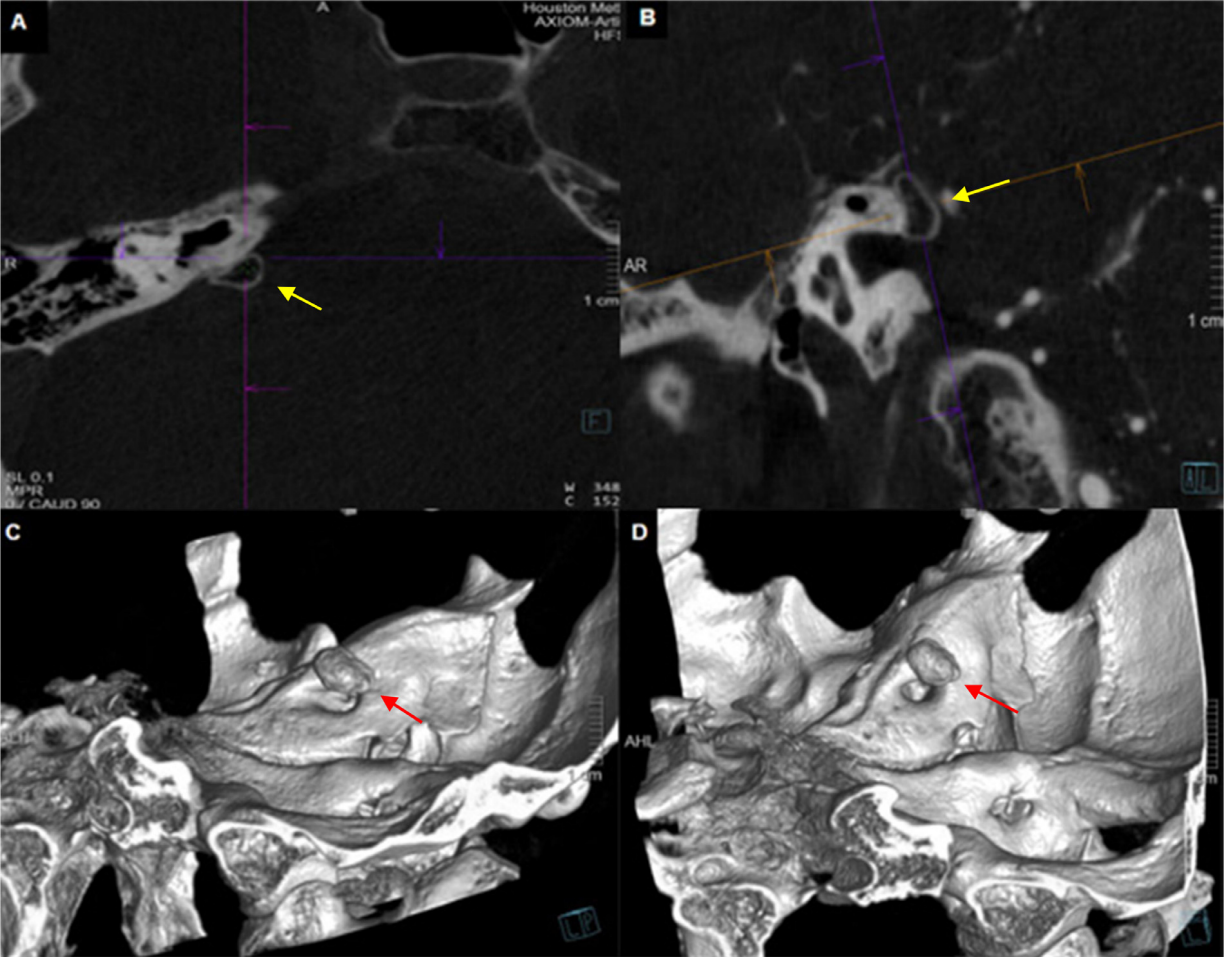
(A) Axial cone beam CT reveals a well-defined 5 mm lesion abutting the internal auditory canal, characterized by a hypodense center surrounded by a hyperdense rim. (B) Coronal cone beam CT illustrates its relationship with the internal auditory canal. (C and D) Tridimensional (3D) reconstruction of the Cone Beam CT illustrates the lesion and its relationship with the internal auditory canal.

A CT scan of the head was negative for intracranial hemorrhage (Fig. 1). MRI revealed a lesion in the CPA that appeared hyperintense in both T1 and T2 sequences with a hypointense halo surrounding both sequences. These MRI findings suggested a partially thrombosed aneurysm of the right anterior inferior cerebellar artery (AICA) or a lipoma as potential differential diagnoses (Fig. 1). Additionally, a cerebral angiography showed no evidence of aneurysms (Fig. 2). As part of the cerebral angiography, 3-dimensional imaging and CBCT were performed, revealing a well-defined lesion abutting the internal auditory canal (Fig. 3).

## Discussion

While multiple potential etiologies can affect the cerebellopontine angle (CPA) and the temporal bone, tumors affecting the CPA are rare, constituting only 5% to 10% of intracranial tumors, with a low malignancy potential [1]. Computed tomography (CT) scans, typically high-resolution multi-detector computed tomography (MDCT), magnetic resonance imaging (MRI), and in some cases cerebral angiography, all play a role in the diagnostic armamentarium during the imaging workup for lesions affecting these anatomical structures [2]. Nonetheless, there is no single best diagnostic imaging modality; therefore, selecting between the multiple options depends on the particularities of every case.

Our patient presented with progressive, sudden onset, severe headaches associated with changes in intensity with position, accompanied by tinnitus and dizziness. An initial CT scan of the head ruled out intracranial hemorrhage, after which an MRI was done. The MRI showed a 5 mm, round lesion located in the CPA, in intimate contact with the temporal bone, that was hyperintense in T1 and T2 surrounded by hypointense halo, suggestive of either an anterior inferior cerebellar artery (AICA) thrombosed aneurysm or a lipoma [3], [4], [5].

AICA aneurysms are rare, accounting for up to 1.5% of all brain aneurysms, and predominantly affect adult women without risk factors; however, their timely treatment remains paramount, as complications like the subarachnoid hemorrhage from their rupture can increase the patient's morbidity and mortality [5], [6]. Therefore, we decided to continue the diagnostic workup with an angiography.

After the cerebral angiography ruled out an aneurysm, a high-resolution multi-detector computed tomography (MDCT) was planned to assess the temporal bone morphology more accurately; nonetheless, a cone beam computed tomography (CBCT) was used instead since the patient was already in the angiography suite. This allowed us to perform the CBCT as soon as the angiographic procedure finished, utilizing the onsite C-arm.

CBCT has better spatial resolution than MDCT, with significantly lower radiation doses, and is especially suited for dense structures like the temporal bone [7]. A few studies have compared the morphologic concordance between CBCT and MDCT in the imaging of the temporal bone and concluded that both allow for equivalent and excellent imaging results based on comparisons between measurements from anatomical landmarks from the middle and inner ear regions [7], [8]. Three-dimensional (3D) reconstructions of the temporal bone from images acquired via CBCT are also considered as reliable as 3D reconstructions from conventional CT [9].

In our case, the enhanced spatial resolution provided by CBCT was crucial in identifying that our patient had a well-defined lesion close to the internal auditory canal. The 3D reconstruction assisted in evaluating the morphology of the lesion and its proximity to the petrous segment of the bone, leading us to conclude that it is most likely an exostosis of the temporal bone.

Despite the potential advantages of CBCT over MDCT, its adoption as an alternative diagnostic imaging technique in the workup of lesions affecting the CPA and the temporal bone remains limited. Consequently, we recommend considering CBCT as an alternative to MDCT in the diagnostic workup of these lesions, particularly in patients undergoing cerebral angiography.

## Conclusions

Our case emphasizes the diagnostic challenges presented by lesions in the cerebellopontine angle (CPA) and temporal bone. Multiple imaging modalities complement each other, but we propose that cone beam CT (CBCT) should be considered among the options to identify these lesions. While computed tomography (CT) and magnetic resonance imaging (MRI) are commonly used, our experience highlights the importance of cone beam CT (CBCT) for its superior resolution and lower radiation doses, particularly beneficial for dense structures like the temporal bone. Further research and documentation of CBCT´s efficacy is necessary to enhance diagnostic accuracy and patient care in neuroradiology.

## Patient consent

I confirm that the patient has provided written informed consent for the subject matter related to the case mentioned above ("Utility of cone beam computed tomography for rare temporal bone lesions: A case report") to appear in a journal article or to be used for a thesis or presentation.
